# Integrated Bio-Search: challenges and trends for the integration, search and comprehensive processing of biological information

**DOI:** 10.1186/1471-2105-15-S1-S2

**Published:** 2014-01-10

**Authors:** Marco Masseroli, Barend Mons, Erik Bongcam-Rudloff, Stefano Ceri, Alexander Kel, François Rechenmann, Frederique Lisacek, Paolo Romano

**Affiliations:** 1 Dipartimento di Elettronica, Informazione e Bioingegneria, Politecnico di Milano, Milano, 20133, Italy; 2 Leiden University Medical Center, Leiden, 2333 ZA, The Netherlands; 3 Netherlands Bioinformatics Center, Nijmegen, 6500 HB, The Netherlands; 4 Department of Animal Breeding and Genetics, SLU-Global Bioinformatics Centre, Swedish University of Agricultural Sciences, Uppsala, 75124, Sweden; 5 Department of Immunology, Genetics and Pathology, Uppsala University, Uppsala, 75108, Sweden; 6 GeneXplain GmbH, Wolfenbüttel, 38302, Germany; 7 Institute of Chemical Biology and Fundamental Medicine SBRAS, Novosibirsk, 630090, Russia; 8 Inria Grenoble Rhône-Alpes, Saint-Ismier cedex, 38334 France; 9 Proteome Informatics Group, SIB Swiss Institute of Bioinformatics, 1211 Geneva 4, Switzerland; 10 Section of Biology, University of Geneva, 1211 Geneva 4, Switzerland; 11 Biopolymers and Proteomics, IRCCS AOU San Martino IST, Genoa, 16132, Italy

## Abstract

Many efforts exist to design and implement approaches and tools for data capture, integration and analysis in the life sciences. Challenges are not only the heterogeneity, size and distribution of information sources, but also the danger of producing too many solutions for the same problem. Methodological, technological, infrastructural and social aspects appear to be essential for the development of a new generation of best practices and tools. In this paper, we analyse and discuss these aspects from different perspectives, by extending some of the ideas that arose during the NETTAB 2012 Workshop, making reference especially to the European context.

First, **relevance of using data and software models **for the management and analysis of biological data is stressed. Second, **some of the most relevant community achievements **of the recent years, which should be taken as a starting point for future efforts in this research domain, are presented. Third, **some of the main outstanding issues, challenges and trends **are analysed. The challenges related to the tendency to fund and create large scale international research infrastructures and public-private partnerships in order to address the complex challenges of data intensive science are especially discussed. The needs and opportunities of Genomic Computing (the integration, search and display of genomic information at a very specific level, e.g. at the level of a single DNA region) are then considered.

In the current data and network-driven era, social aspects can become crucial bottlenecks. **How these may best be tackled to unleash the technical abilities **for effective data integration and validation efforts is then discussed. Especially the apparent lack of incentives for already overwhelmed researchers appears to be a limitation for sharing information and knowledge with other scientists. We point out as well how the bioinformatics market is growing at an unprecedented speed due to the impact that new powerful *in silico *analysis promises to have on better diagnosis, prognosis, drug discovery and treatment, towards personalized medicine. An open business model for bioinformatics, which appears to be able to reduce undue duplication of efforts and support the increased reuse of valuable data sets, tools and platforms, is finally discussed.

## Background

The "bio-data deluge", intrinsically caused by high-throughput technologies, is currently providing scientists with very rich, but also almost unmanageable information. Techniques like Next-Generation Sequencing (NGS), only to mention the most widespread, generate data on an unprecedented scale and are now driving the generation of knowledge in all areas of the life sciences to new dimensions [1].

The abundant information sources that are being created are not fully exploited because of the difficulties in finding, selecting, extracting and integrating the most appropriate information to address a biological question. Moreover, typical questions are increasingly complex and frequently require the simultaneous analyses of a great variety of data from multiple heterogeneous information domains and resources; they often make reference to different organisms' levels, e.g., whole organs, tissues, cells, and biomolecular entities. Consequently, the life science community urgently needs new and improved approaches to facilitate data management and analysis, which need the integration of data resources [2]. We loosely define this activity as "*Integrated Bio-Search*". **Integrated Bio-Search includes, then, all aspects relating to technologies, methods, architectures, systems, and applications for searching, retrieving, integrating and analyzing data, information and knowledge that are required to answer complex bio-medical-molecular questions, by means of the most appropriate infrastructures, services and tools **[3]. Although we see the above aspects as an integral part of good "data stewardship", we explicitly exclude from this paper other significant data stewardship challenges, like data storage and accessibility and related tools.

Available computational infrastructures support only part of the tasks required to answer questions in modern biology. Currently, scientists need to laboriously explore available sources via multiple and heterogeneous search services, compute data analyses via various Web interfaces to the many valuable, but not interoperable, tools accessible on the Internet, and finally struggle in combining selected information answering the original question. This situation partly arises as a consequence of too many individuals developing their own solutions without synergistically contributing to sharing initiatives. Furthermore, the human-centric nature of most bioinformatics resources is yet another source of complication in addressing questions in veterinary or plant sciences.

Structural improvements in finding, assessing and combining multiple inter-linked data and algorithmic sources have the potential to reshape the scenario of current bioinformatics applications, going way beyond the capabilities of conventional tools, Web Services and existing search engines. This scenario presents new methodological and technological challenges that we review in this paper. Our major aims are to ensure that there is at least awareness of the major ongoing community driven efforts and to stimulate convergent evolution of best practices. We are convinced that **solving data integration and automatic extraction problems requires formal models for data, information, tools and workflows**. It also needs **radically innovative solutions and some discipline**; they include the use of universal identifiers - such as computer processable Uniform Resource Identifiers (URIs) and Universally Unique Identifiers (UUIDs) - to refer to concepts, proper study capture frameworks, Semantic Web approaches, efficient pre-indexing, partial or approximate value matching, rank aggregation, continuous or push-based search and intelligent alerts, exploratory methods and context-aware paradigms, collaborative and social efforts, as well as building new efficient information retrieval approaches, based on automation of persistent and reusable workflows. The power of formalization and modelling of all these aspects is crucial for their wide reusability and, thus, for a widespread adoption in the community. In the following sections we focus in particular on biological data and process modelling, formats, standards, ontologies, computational infrastructures and technologies, as well as on data and information indexing and search.

## Formal modeling in life sciences

The need for formal approaches is not less important in biology than in the physical sciences. Formalization brings several critical advantages. First of all, it allows for non-ambiguous definition of concepts. Think about the multiple acceptations of the term "gene" in narrative. Embedded in a semantic network or in a database schema, the class "gene" acquires a unique definition. Supplemented with a definition written in narrative language, it offers an efficient support for person-to-person communication and at the same time for computer-based implementation. Then, formal models may offer prediction capabilities. Differential equation systems are known to be a powerful and effective way to represent dynamical systems and to compute the evolution of their variables over time through their simulation. They are intensively used in mathematical ecology to study and predict the evolution of population sizes in ecosystems. In other cases, however, the need to provide quantitative values to the parameters of their equations presently limits the use of differential systems in biology. It is typically the case when studying gene interaction networks, for which quantitative data are still lacking. However, in that case, other types of formal models, such as Boolean equations or semi-qualitative equations, may be used.

As a consequence of the scarcity of mathematically expressed laws describing the complexity of biological systems, computer science may provide key elements to address the increasing need for formal modeling in the life sciences. This is demonstrated by the growing importance of bioinformatics, algorithms and software. Computer science does indeed bring to biology numerous specific modeling formalisms, relying on discrete mathematics, theory of languages, logic, and knowledge representation.

Databases, as opposed to files, are the very first example of such contribution. Database design relies on describing the domain to be covered and formalizing entities and their relationships. Simultaneously, it directly defines the integration of many heterogeneous bio-data to enable comprehensive analysis. Today, the Unified Modeling Language (UML) [4] and its derivatives are often used for this first step, resulting in a documented diagram that can be read and interpreted by humans on the one hand and leads to implementation, typically in relational database management systems, on the other. Shifting from data storage in tabular files to data structuring in a database is thus already a quantum leap into formalization and disambiguation, offering simultaneously powerful retrieval, query and analysis facilities.

Computer science provides many other modeling tools, which have no mathematical equivalent. Production rules can be used to represent methodological expert knowledge. This expertise can be integrated in complex data analysis pipelines in which input data and intermediary results are used to select, chain-up and parameterize appropriate methods. Formal grammars are an elegant solution to simulating morphogenesis. Multi-agent models also describe and simulate complex interacting entities. Boolean equations describe basic gene interaction networks. A strong advantage of all these formalisms is their effective and efficient implementation as operational software.

Conversely, biology has a lot to offer to computer science. An example of the reciprocal benefits of both disciplines is the "associative" power that computers have now gained, beyond formal logics, through dynamic concept webs. This allows computers to go "beyond the obvious" and make "new" predictions that were too complex or inaccessible by human reading and synthesis. Furthermore, implicit and indirect associations in highly complex concept webs can now be meaningfully exposed by computer processing and actually guide the human *in cerebro *discovery process. In fact, computers can work more closely to the way the human mind works [5].

Computer science meets the modeling and integration requirements of biology so tightly that it will soon play the same role in biology as pure mathematics played and still plays in physics. Interestingly, the evolution of bioinformatics method validation illustrates this growing interconnection. In computer terms, criteria for assessing a piece of software are intrinsic qualities spanning algorithmic soundness, running time or statistical behaviour. Technically speaking, these criteria can be considered sufficient for theoretically validating a method and its underlying model. In biological applications however, the importance of benchmarking with reference or test datasets remains essential. In the early days of sequence analysis, artificial sequence data sets or unfiltered database search results with loose keywords have been used in a number of bioinformatics method papers as technical validations for new algorithms, thereby remotely solving any related biological question. The introduction of Receiver Operating Characteristic (ROC) analysis [6] in evaluating predictive models and the quasi-systematic computation of sensitivity/specificity measures were a first step towards reducing this validation gap between the two disciplines. The rising of "-omics" data bridged the definition of models and their validation. Now, many models are designed to analyse large-scale data, and validated "in the process" through the production of interpretable results. For instance, both the number of identified proteins and the rigorous underlying statistical models are central to validating mass spectrometry data analysis methods developed for the purpose of identifying proteins in a proteomics experiment. Data and method validations have become inseparable.

In summary, computer science can provide modeling of four different levels minimally needed to:

1. formally represent relatively simple scientific assertions,

2. represent networks of such assertions and associations in pathway format,

3. quantitatively approximate the dynamics in such pathways,

4. describe the actual scientific workflows used to capture, process, integrate, analyse and model data.

In the following sections, we give illustrative, though not comprehensive, arguments to support our vision, shaped by our respective experience. The aim is to demonstrate in principle that it makes sense to treat each identifiable artifact produced in research, which might be "reusable" as a research object, and annotate it with sufficient metadata and provenance to actually make it sustainably accessible and reusable.

## Early community efforts and achievements

### Standards arising from bioinformatics research for human biology

It is now sufficiently clear that Web Services can play an effective role in this context, but, in order for them to achieve a widespread adoption, standards must be defined for the choice of Web Service technology, as well for semantically annotating both service functions and the data exchanged; furthermore, a mechanism for discovering services is needed. However, experience is now overwhelming that the real standards used in biology emerge from the community.

One of the pioneering projects trying to address these problems has been the *EMBRACE *EU FP6 funded project. The goal of this project was to enable biomedical research in the "-omics" era just before the NGS tsunami. The major products that resulted from this five years long project were:

*1. EDAM ontology *[7], that covers common bioinformatics operations, topics and types of data, including identifiers and formats. It comprises concepts that are shared within the bioinformatics community and apply to semantic annotation.

*2. EMBRACE Web Service registry *[8], a collection of life science Web Services with built-in service testing and a prelude to the internationally supported BioCatalogue system [9].

*3. BioXSD *[10], a common exchange format for basic bioinformatics data, was designed and implemented.

**Figure 1:**
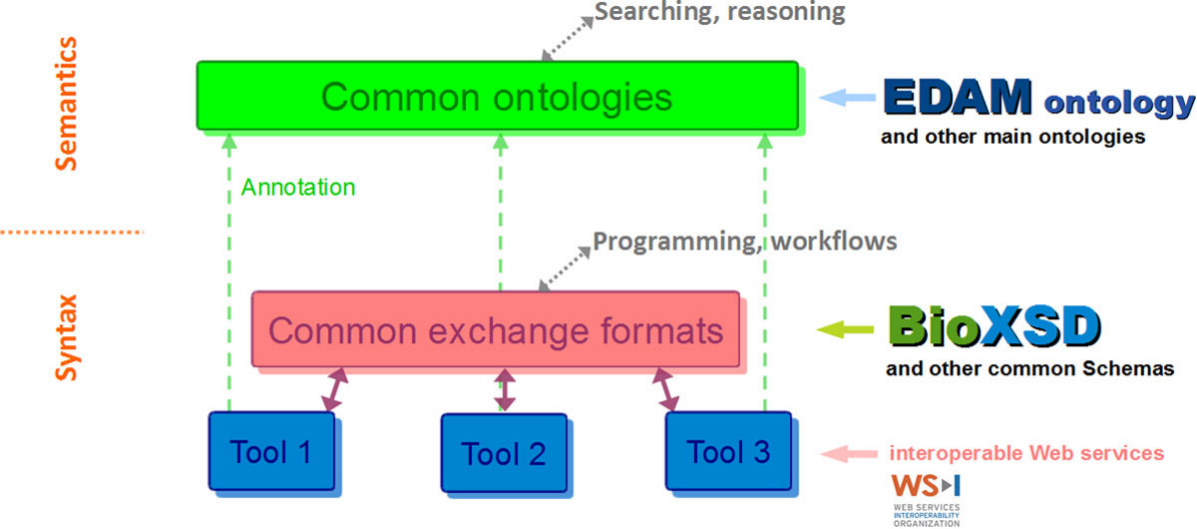
**Intended interactions between EDAM ontology and BioXSD schema**. The semantics layer supports searching by end users, as well as automated reasoning. Both these tasks leverage shared ontologies. The syntax layer supports actual interoperability between tools, as well as programmatic access; both tasks leveraging common exchange formats and schema. The two layers are made consistent by a proper ontology based annotation of data and services.

Intended interactions between EDAM and BioXSD are shown in Figure 1.

Another project funded in EU FP7, was *GEN2PHEN *[11]; it pioneered the data, database and workflow challenges in collecting and sharing human genotype and phenotype data.

Finally, *Open PHACTS *[12] is a major knowledge management effort launched under the Innovative Medicine Initiative (IMI) framework. It is widely supported by pharmaceutical companies, but it also moves beyond the pharmaceutical realm. It is the first project that yielded a widely used infrastructure based on Semantic Web technology. The project attracted multiple associated partners and the beta version had more than one million hits. A rapidly increasing number of public and private partners adapt their services to use the content and the data model (described in Resource Description Framework (RDF) with rich provenance) of Open PHACTS. The technology developed by this project is generic and will increasingly be adopted in other fields of the life sciences.

### Beyond the human species

A variety of bioinformatics software solutions, analytical methods and common procedures and standards purposely devoted to the human species are available. Conversely, non-human life science communities exist with different degrees of scientific coordination, and some areas have already agreed on ontologies and common procedures and standards. But only few efforts have gone into the wider task of harmonizing the research efforts of these communities.

By adapting existing technologies from the field of human bioinformatics and developing them further, it is possible to build working infrastructures for bioinformatics within the non-human fields of life sciences. AllBio [13], a EU FP7 KBBE project, coordinates efforts to make the human genome related technologies operational in the fields of microbial, plant and livestock. Partners in AllBio collaborate on subprojects such as the design of ontologies for data and methods, and the choice of common interoperability standards.

### Cyber-infrastructures

The role of grassroots communities in creating and enforcing standards was highlighted in examples cited above. Integration is a matter of standardization, and effective standardization requires the common adoption of methods, models and tools. Nowadays, communities can best interact through ICT infrastructures to reduce and overcome space and time limitations. The European Strategic Forum on Research Infrastructures (ESFRI) and its related Research Infrastructures, many of which are now being implemented, is a perfect witness of this need and perspective. In 2012, the European Commission launched a call to all research communities to identify topics requiring integration in national research infrastructures: 547 proposals representing 246 topics were submitted. A refined selection of 135 topics with high potential and merit for future Horizon 2020 actions, 35 of which were from the Biological and Medical Sciences (BMS) area, were listed in the final report [14]. Among them, topics listed in Table 1 are worth mentioning; they are related to data integration and search, and clearly reflect the need for the outcomes of initiatives mentioned above. In addition, virtually all projects funded in EU FP6 under the ESFRI and IMI schemes have a (big) data component and call for a higher level and more formal collaboration.

**Table 1:** Main Biological and Medical Science topics with high potential and merit for future Horizon 2020 actions.

High potential Biological and Medical Science topics
Integrated disease and phenotype ontologies and supporting tools
Molecular profile reference databases for cells and tissues
European infrastructure for genome research
European animal genomics and phenomics infrastructure
An integrating activity for fish genome resources
Trans-national infrastructure for plant genomic science
European proteomics research infrastructure
Integration of national non mammalian model animal facilities on the European level
European primate network: maintaining and developing best practice, staff education and international standards in biological and biomedical research
Cyber-infrastructure for farmed and companion livestock
An integrated technology platform for high-throughput, multi-level phenotyping research to design robust farm animals for tomorrow
Network of animal biological resources centers
Aquaculture infrastructures for excellence in EU fish research
European network of high containment animal facilities to improve control of livestock transboundary and zoonotic infectious diseases
European seed bank research infrastructure
Forest tree genetic resources, a pan-European patrimony to be maintained and developed at the benefit of the scientific community
Improved access of the scientific community to collections of non pathogenic, pathogenic, emerging and clinical human/animal virus isolates (including fish and arthropods) up to biohazard risk group 4
Facilities, resources and services for mining the nature and relevance of biocide resistance
Pan-European resource for gene transfer vectors towards clinical application

The *BioMedBridges *project [15] was funded to investigate the creation of bridges between the ESFRI and other research infrastructures in order to prevent the development of data silos and non-interoperable tools. Currently, more than 10 ESFRI projects started to coordinate efforts in BioMedBridges. This shows a natural tendency of countries and international projects towards sharing the burden of data stewardship and management. Optimal sharing of best practices, data sets, tools and infrastructures within disciplines of biology, but notably also across the human, animal, plant, nutrition and biotechnology disciplines, will be driven by scarcity and scalability of resources. Most notably, *ELIXIR *[16], with the tagline "data for life", is a candidate for a coordinating role. Communities should monitor the development of ELIXIR and, where possible, align, coordinate and share local expertise with this international environment.

## Major outstanding issues, challenges and trends

### New integration and search challenges for Genomic Computing

Management of data generated by NGS technologies is a paradigmatic illustration of the so-called "big data" challenge. The integration and search of genomic information is a problem of its own and serves as a leading example, as we expect comparable quantum leap developments in other "-omics" technologies and imaging as well. Current formats and standards for the representation of NGS data are inadequate to support efficient and high-level search of information, as they are mostly concerned with the encoding of DNA-related information and not with its use within query and search systems. The interoperability standards, such as the Distributed Annotation System (DAS) [17], appear similarly inadequate to efficiently support interoperability at the level that is required by the breadth and complexity of exchanged information. In reality, the "data deluge" generated by NGS technologies has not been matched by corresponding progress in data query, integration, search and analysis, thus creating a gap in the potential use of NGS data.

Covering the gap is not easy. On the one hand, data integration is known to be a hard problem in all fields, as it requires coping first with different data semantics across data sources and then with efficient data sharing in the presence of replication and errors. In NGS, problems are amplified by the lack of standards for exposing data semantics at a level where it can be well understood and appreciated. Indeed, while the bioinformatics community has made enormous progresses in the description of several general-purpose ontological sources, similar attention has not been given to a high-level description of experimental and annotation data. Several databases and ontologies, as well as tools, exist for describing the general features of experimental data [18,19], or for connecting to annotations and displaying the corresponding DNA regions [20], but little emphasis has been put on describing experimental results going beyond data formatting. Hence, the focus on specific DNA regions of experimental data sets, in order to "read" the experiment from a particular biological or medical perspective, is not adequately covered; but such capabilities are key ingredients to the support of biological research and personalized medicine. Indeed, the most important data integration involves the human genome, which description undergoes frequent updates of alignment references. his has been discussed for a period of over a year by a group of engineers and computer scientists from the Politecnico di Milano (Polimi) with biologists and bioinformaticians of the Istituto Europeo di Oncologia - Istituto Italiano di Tecnologia (IEO-IIT). The conclusion of this group is that the field requires a revolutionary, data-centred approach [21]. Practices established in various research laboratories, involving data alignment and data analysis pipelines, cannot be easily interfered with. Yet, there is a need for interpreting experimental data at a high level of abstraction, in terms of specific properties of genomic regions. Such interpretation is facilitated by the presence of few well-understood physical data formats (e.g. FASTQ, SAM, BAM, BED, bigWig, etc. [22]) that are suitable for data extraction via simple wrapping technologies. Thus, it is possible to extract region-aware data in high-level format from experimental or annotation data sets (e.g. height, width and probability of peaks in a ChIP-Seq experiment which satisfy a given threshold of extraction, relative to the genome region where peaks are expressed). Such information is much more compact and semantically rich than the one that can be expressed in the BAM, BED or bigWig data formats. Furthermore, it can be processed by high-level programming languages.

A joint Polimi/IEO-IIT effort is ongoing towards the definition of a "genometric data model" and a "genometric query language" which can be used to describe the information contained within each data set. The data model associates a semi-structured collection of metadata with each experimental data file; moreover, each data set is transformed into genomic regions, each having coordinates relative to a reference assembly and associated with specific data (e.g. describing mutations, gene expressions, transcription sites, etc.). The genometric query language is capable of high-level operations such as comparing experiments, extracting their most interesting regions and mapping a given region description to another within the "genometric space". It thus provides a good starting point both for pattern-based queries, e.g. by extracting experiments or regions that exhibit specific data patterns, and for data analysis, e.g. by constructing region-to-region or experiment-to-experiment networks highlighting their similarity or relationships. While this research project is not the only one dealing with high-level query languages for NGS data (e.g. see [23]), it aims at preserving the way in which data sets are produced and primarily analysed in experimental laboratories, and operates on top of these primary analyses.

A high-level description of genome information is the starting point of content-based indexing and search. Genomic information can be indexed by information content and significance in much the same way Web pages are indexed by word content and significance. If research centres make their NGS data available in some form of Web interface (ranging from basic DAS 1.6 or DAS 2.0 versions, up to direct exposure of an Application Programming Interface (API) to genometric queries), it will be technically possible to implement a process very similar to Web crawling, extending access to all the research centres who agree to share (part of) their NGS data.

By coupling indexing to crawling, we come to a vision of the "Web of Genomes" as a powerful infrastructure supporting the future of Genomic Computing. We can initially assume simple search patterns, such as finding experimental data related either to a given pathology (based on metadata) or to a panel of mutations localized on DNA regions (based on region information). Search patterns may then grow in complexity, up to encompassing similarity search with specific genome regions, which characterize a given experiment. The identified and extracted insights from highly variable original data files could then be stored as associations in a computer readable and interoperable format, such as the provenance-rich RDF, i.e. as nanopublications (see next section), that would seamlessly connect them to all core legacy information in the same format.

How far is this vision? We still need a number of well-traced technological achievements, based on well-established practices that have been already applied to other fields and primarily to the Web as we know it. This vision arises from strong previous expertise in generic data and Web management. It can progressively turn into reality through coordination, co-operation and support towards reshaping the field of data search and integration for Genomic Computing.

### From data to information

As soon as data sets have undergone a first level of pre-processing and analytics, even before the actual biological interpretation and knowledge discovery start, the "associations" and "assertions" about how concepts addressed in the data sets may relate to each other become apparent. This can range from simple associations, such as co-location or co-expression, to entirely fleshed out assertions about how a given post-translational modification influences the 3D structure of a protein. Such associations, in essence, follow the model of "subject-predicate-object" (SPO) triples, such as those operated in RDF. Notably, certain assertions need more than one SPO to become meaningful. For instance, a single nucleotide polymorphism (SNP) in a given position (triple 1) and in a given species (triple 2) may cause a certain protein change (triple 3). Therefore, in many cases a small named graph is needed to form a minimal assertion. Following this principle, a "nanopublication" has been defined as the smallest possible meaningful assertion published in RDF [24]. It also relates to the concept of "Research Objects" [25], which has been introduced in order to capture the need for more formal modeling in biology in the broadest sense. A Research Object is an aggregation object that bundles together experimental resources that are essential to a computational scientific study or investigation. If we define a research object, *ad interim *for the purposes of this position paper, as "any identifiable artifact produced in the activities of research and formatted for computational studies", such research objects cover formal data models of level 1 (such as nanopublications and micropublications), level 2 (such as a formal pathway in, for instance, WikiPathways), level 3 (any system biology qualitative model) and level 4 (for instance well documented and reusable workflows).

All concepts in a nanopublication graph should ideally refer to a well established vocabulary, so that linking to ontological knowledge is possible and computers understand exactly what is meant by each URI in the graph. It is important that nanopublications can also be expressed in a human readable language, based on a correct linking of the URIs in the graph and the terms used in a narrative (in different languages) of the concepts in question. Next, the nanopublication needs provenance to be placed in context. Not only minimal information about the conditions under which the assertion has emerged and those under which it is considered "true", but also all other metadata that are usually associated with a classical narrative research article (such as authors, publisher, etc.) should be associated with the nanopublication. In fully compliant nanopublications, these parts of the connected graphs forming the entire nanopublication are also modeled in RDF (see guidelines and examples at [26]).

Nanopublications have now been created from different data types, such as locus specific databases [27], the Fantom 5 data set, GWAS data [28], chemistry and pharmaceutical databases [29], UniProt and neXtProt. In principle, each data source containing assertional information can be republished in this format, which is both machine interoperable and human readable, with relatively limited effort, without distorting neither the original data format nor the legacy database. Nanopublications can also be re-created by text mining, although they suffer from the same challenges as all text mining approaches [30]. An increasing number of narrative sources (including PubMed) are now being "nanopublished", i.e. published as nanopublications, and major international publishers are investigating how they can expose the scientific conclusions and evidences contained in their narrative collections in this format.

## Expanding collaborative efforts and broadening communities

In the previous sections, we have highlighted some technologies and methodologies that can efficiently support new data integration and search challenges set by NGS and the development of new high-throughput equipment. We have especially stressed the role of modelling, standardization, and interoperability. We also hinted at addressing methodological and technological outstanding issues through the expansion of collaborative efforts. As in other fields, community efforts, such as data annotation and curation, are progressively enabled by the growing support of social information and communication technologies. The technical environments that are available for community annotation, data publishing and integration play an increasingly important role in the life sciences [31-34]. Yet, some factors are still limiting the possible valuable contributions arising from social efforts. Here, some of these factors are shortly discussed, focusing on those that restrain the participation of scientists to bio-data integration, mining and validation. In particular, we identified two major difficulties. First, scientists appear to currently lack the motivation to contribute positively to annotation in databases or knowledge bases. Second, valuable work done by authors who do not produce *de novo *data, but carefully select data from repositories for reanalysis, is poorly acknowledged. The following sheds some light on these questions and the various possible answers brought by communities with different social roles.

### Data curation: from ignoring to cooperative

Many of us search or browse bioinformatics online resources. While doing so, we occasionally activate a link that unexpectedly breaks or points to absurd content. We mentally complain about it, but we usually ignore it and resume browsing. Some of us do spend the couple of minutes necessary to report this broken or mistaken link to the development team (if still in existence) and thereby spare the trouble to other users. In this case, "contributing" is pointing out errors but not solving issues. Indeed, too few of us envisage on-line resources as community wealth to which contribution would mean definite improvement and added value, i.e. a form of curation, which benefits all.

The activity of biological data curation has evolved over the years to a point where there is now an organised International Society of Biocuration [35] within which the question of community-based curation is debated and promoted, among other themes. The need for a coordinated action in this domain was emphasised for instance when the Swiss-Prot team introduced in 2007 the "adopt a protein" scheme [36], encouraging specialists of a given protein to oversee the update of the corresponding UniProtKB/Swiss-Prot entry. As it seems, scientists are not born protein adopters and the initiative could not be sustained. In the same period, a more sophisticated wiki based attempt was made in *WikiProteins*: the paper calling for a million minds [Mons 2008] from 2008 has meanwhile collected more than 120 citations, but is not in line with the number of community annotations in WikiProteins, and the attempt was discontinued. Other wiki-based approaches, such as the *GeneWiki *(in the context of WikiPedia) [32] and for instance *WikiPathways *[33], have met with slightly more traffic, but to the best of our knowledge the only community annotation effort that really took off to a level of satisfaction is *ChemSpider *[37]. However, with these lessons learned, an alternative invitation to contribute was devised a few years later in the human protein-centric knowledge platform *neXtProt *[38]. The neXtProt scheme promotes users' participation through the specific input of a selected network of specialists. Experts contribute by submitting experimental data sets and defining metrics for quality filtering in agreement with the neXtProt team. Very recently, curated associations on, for instance, Post Translational Modifications from NextProt have been formatted as nanopublications; this will allow the community contributions to certain snippets of information to be fully recognized (see next section).

In essence, biological data curation history tends to show that direct contribution may not be the ultimate strategy for gathering quality information and attracting potential contributors when it is limited to the addition of comments or facts in a Web page. Instead, guided input, so as to capture and shape information upon criteria that were previously and collectively agreed upon, seems more of a realistic approach. Future tools should rely on social interfaces encouraging users' cooperation in a constructive and targeted manner. Some efforts have already been made in this direction, e.g. for collaborative ontology development [39] and for interactive knowledge capture by means of Semantic Web technologies [40]. Yet, this important future area of scientific contribution suffers from the same roadblock as the "data-based science" discussed before. Unless a culture develops where these contributions (when measured perfectly) influence the career of the next generation of scientists, community contributions will always be limited to the "altruistic few" [41].

### Exchange, access, provenance and reward models

An outstanding issue is the social award system and the perceptions prevailing around data sharing. In white papers advocating data sharing, authors usually emphasise technical challenges rather than the actual process of data sharing, although there are as many social challenges associated with actual data sharing as there are technical challenges. Obviously, technical challenges come first: data can only be shared if they are interoperable in format or have been captured with proper metadata attached.

Making data Open Access is clearly not enough; data accessibility and reusability by others than the data generators, is what really matters. As stated in previous sections, reuse of valuable data sets will support e-science discovery processes. In this context, provenance is the key for users planning to include existing data in a meta-analysis. Prior to adding a data set to the analysis mix, an e-scientist needs to evaluate the set, its overall relevance, quality and the underlying methods. For this crucial decision step, the metadata, including rich provenance, are needed.

In many cases, data can be excluded or included from/in an analysis workflow by properly instructed machines. For instance, all data on genes of a given species, e.g. mouse, can be automatically discarded, as long as sufficient provenance is associated with each candidate data set. It is thus very important that the concept "mouse" as *Mus musculus *is associated with a data set based entirely on mouse experiments, and properly referred to with a computer readable identifier. But it is also important that such identifier is at the appropriate position in the metadata fields, or in a RDF graph; this, for instance, to allow for the distinction between an occasional mention of the concept *Mus musculus *in a table or graph, as opposed to the statement "this entire set was generated on 'mouse' experiments". However, this ideal situation is currently far from reality. Even with Digital Object Identifiers (DOIs) for data sets and initiatives such as *FigShare *[42], *Dryad *[43] and the *Research Data Alliance *[44], we will need many years before each valuable data set can be properly judged and interpreted by others than its creators. This becomes even more pertinent in multi-scale modeling and the associated multi-omics and multi-technology data sets that increasingly dominate contemporary biology. It is not enough to "find a data set of potential relevance", because soon there will be too many, or to see some metadata on how the study was performed, although this is a *conditio sine qua non*. For real e-science approaches in biology, we need to see the provenance of each individual data element as it may appear, for instance, as a crucial edge in a graph-based hypothetical discovery interface.

Nanopublications and micropublications are here important. It is clear that entire complex data sets with hundreds of thousands, and sometimes millions, of interesting associations can be published as nanopublications; so, they are no longer lost in a huge number of hyperlinks to remote repositories, but each and every individual association becomes a research object in its own right, and is discoverable by computers and humans alike, all across the Web.

Assuming nanopublications and micropublications can solve the data integration issue, the next challenge is to "build a market" for such small information units. They do not intrinsically carry enough evidence to be trusted. The decision to "trust" them or not is taken on the basis of the source information, the associated methods and the reasoning that led to the claim in question. Again, additional steps (in fact, a form of annotation) are needed to create such computer and human readable units with rich enough provenance. This raises the question of finding the means of improving scientists' motivation to spend extra time on annotation.

Naturally, we lack much of the technical infrastructure that is needed to make this all reality, but, in practical sense, these needs are "easier" to fulfil than breaking through the science ecosystem hurdle to make "preparation for sharing" of data a core activity for every data creator and publisher. In fact, what we need is "desktop publishing" of data and information, very much like today authors carefully pre-format their papers according to guidelines for authors. Modern publishers should become data publishers, as well as narrative publishers, and should assist scientists in the curation and shaping of newly published data sets and of their provenance, much the same as they currently do for narrative.

Especially for sensitive data, both in terms of privacy and competitiveness, a *trusted party status *for the needed data publishing and stewardship infrastructure is a *conditio sine qua non*. Therefore, such a data exchange environment can only be built effectively as a federated and "approved" infrastructure, serving national as well as international data driven projects, and as a public-private partnership.

**Figure 2:**
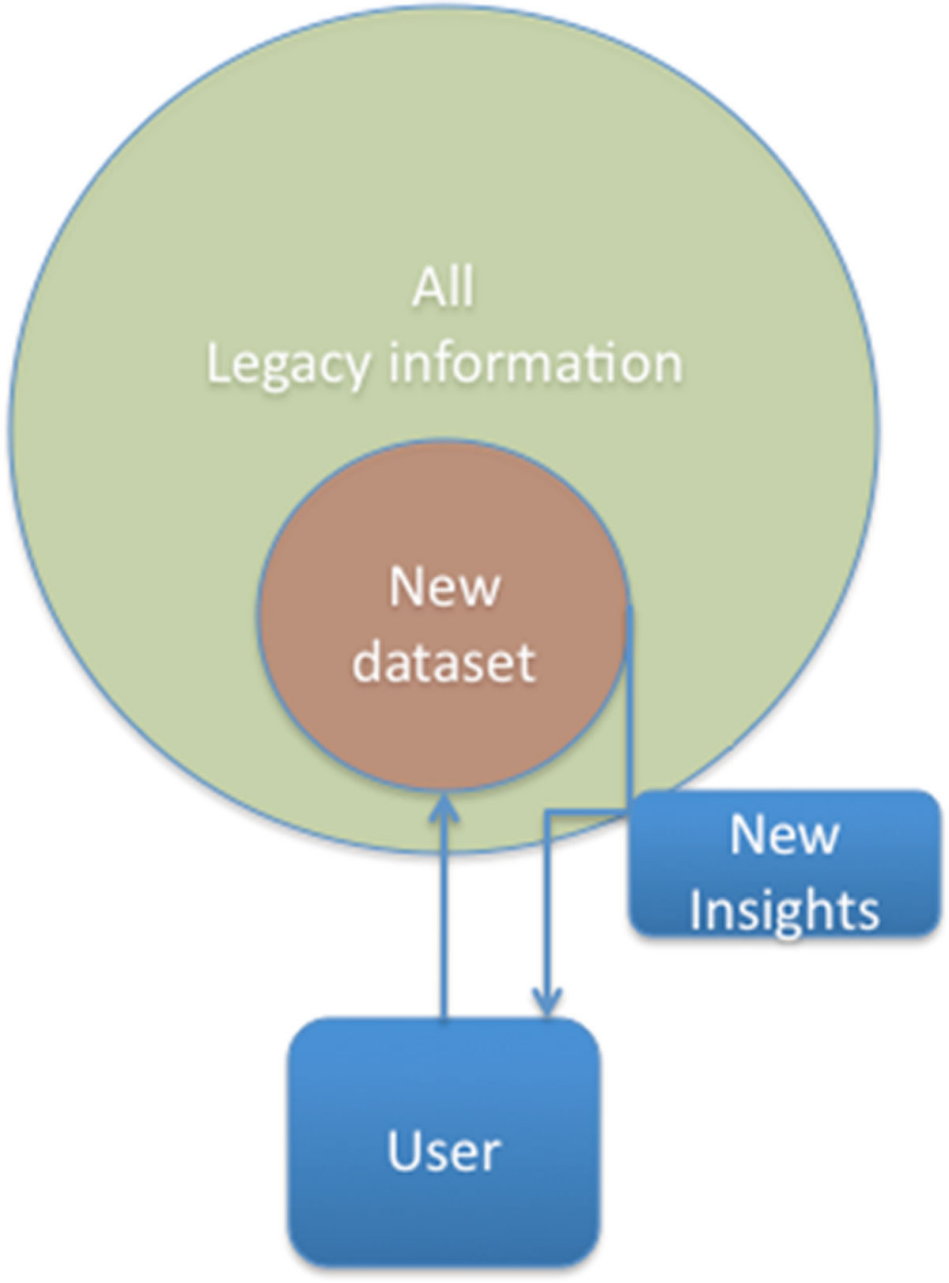
**The basic workflow of data driven science**. The general principle that a data exchange platform should enable and support is depicted. A newly generated data set is combined with other data sets (ideally all core legacy information of relevance) and new insights, including complicated processes, such as multi-omics data integration, multi-scale modeling, computer reasoning and inference, etc., are derived from that data integration and modelling. To this end, users should be allowed to upload their (novel) data and run standard workflows of choice on the combined data.

For purposes of clarity, in Figure 2 we have summarized, and grossly oversimplified, the basic workflow of data driven science. What is needed for e-science is, in fact, a completely new way of publishing, using, searching and reasoning with massive data output, in an open, software-driven, interactive environment.

**Figure 3:**
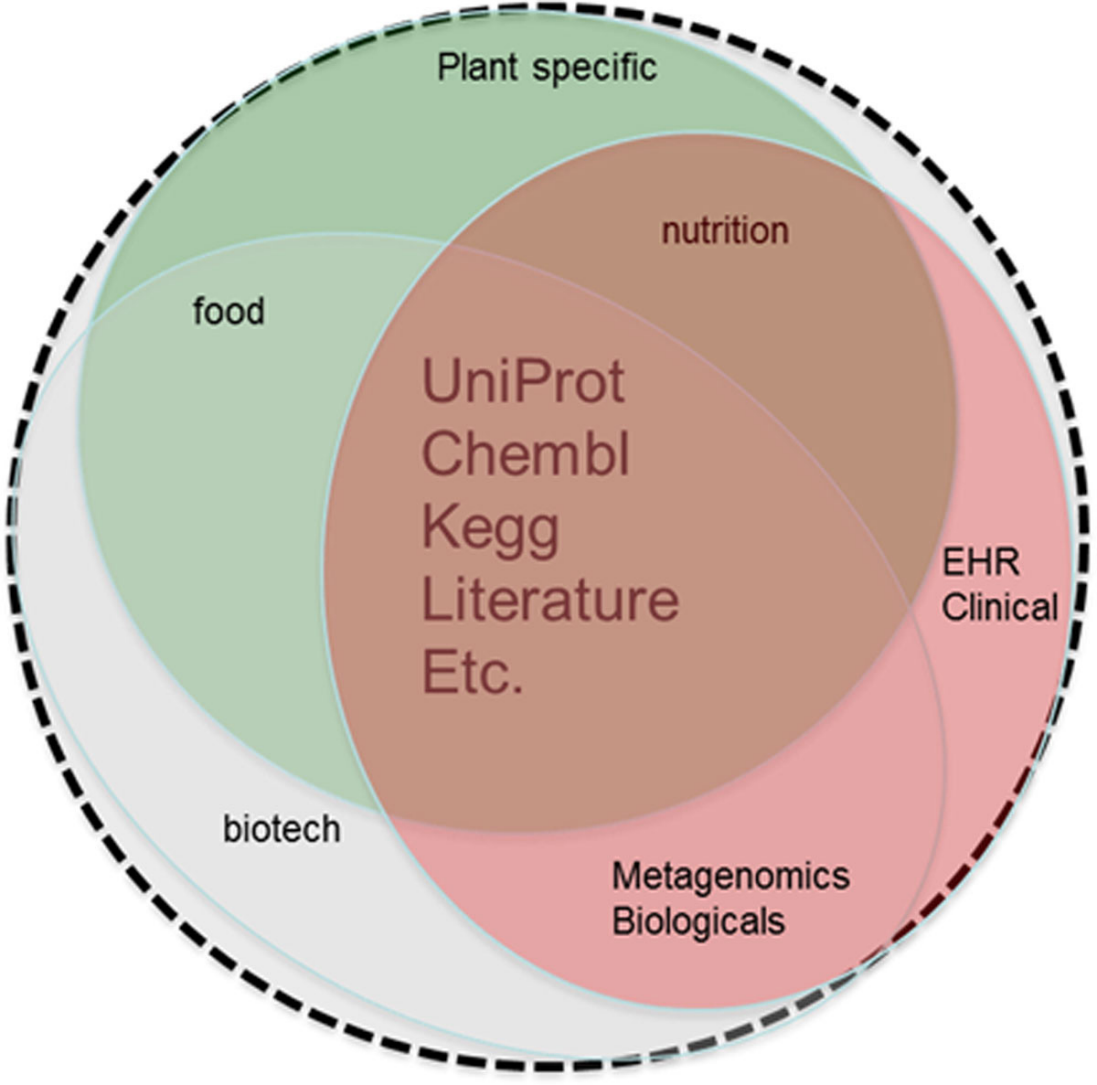
**Integration of scientific data**. Relevant scientific data, that constitute a central core of biological information requested by almost all domains, could be made available in an interoperable format to make their direct integration, comparison and modeling with domain specific data possible.

Relevant scientific data, such as open source publications (e.g. Public Library of Science (PLoS) or BioMed Central (BMC)), individual assertions from closed access publications, abstracts (e.g. PubMed) and relevant legacy data sources (e.g. ChEMBL, UniProt), that constitute a central core of biological information requested by almost all domains, should be made available in an interoperable format to make their direct integration, comparison and modeling with new data possible (Figure 3). Currently, only a small percentage of information in databases, for instance SNP-phenotype associations, can be recovered by text mining from abstracts, or even the entire narrative part, of full text articles. Many of these types of associations are included in tables and figures, which escape ordinary text mining algorithms, and in supplementary data, which are ignored by text mining. It is therefore crucial to move to a situation where massive numbers of associations can be published in a "discoverable" and interoperable format, with proper references to the producers of the data and associated narrative elements in order to allow award of the efforts.

Notably, publishers have already played a role by imposing annotated data submission and, some of them, by being involved in the definition of related standards, e.g., MINSEQE [45] or MIAPE [46] standards that are governing corresponding data repositories: ArrayExpress [47] for MINSEQE or PRIDE [48] for MIAPE. However, a major roadblock at this point in time is that many grant and manuscript reviewers still do not recognise the value of studies that do not entail the production of new experimental data, but only exploit results from data repositories. Without challenging the sustained importance of proving a biological hypothesis with sound experimental data, it should nonetheless be admitted that **validation does not necessarily impose being the creator of the data used as evidence**.

Finally, social hurdles for data sharing are not limited to the conservatism of publishers and funders, which could be overcome hopefully soon. Additionally and more importantly, there is no "scientific" reward for sharing, i.e. acknowledgement of its value as a scientific product. If no mechanisms exist for any *generally acknowledged *reward for sharing and making own data discoverable, well annotated, principally interoperable and citable, a routine of data sharing is not likely to be established. Movements like *Altmetrics *[49] are crucial to raise a discussion and to demonstrate technical feasibility of a fine grained judgement about an individual's contribution to a scientific record. However, until the "reward" reaches a steady and wide acceptance by reviewers, funding bodies and publishers, nothing will change. They have the means to push researchers make a proper data stewardship part of their natural workflow. It is only since recently that we need to take the "reusability" of the data that are being generated into account in the study design. Several funders already require a well-drafted data stewardship plan for any proposal that will generate significant data sets. This practice should be encouraged; proper standards, best practices, guidelines and reward systems should be implemented and made easily findable, so that biologists with little or no affinity with bioinformatics or data sharing can still participate. Only if all these prerequisites for data sharing are in place, the culture may change and a genuine open data exchange culture in the life sciences can be established.

## Business models for bioinformatics

If all the above would be solved properly, we still need a sustainable environment to make this a reliable part of the scientific practice. This means that funding-cycle based approaches alone will not suffice. Several examples exist of crucial resources, used by each and every scientist, that have faced financial crises in periods between solid funding (e.g. Swiss-Prot [50]). Therefore, it seems that the entire system can function only with private and public parties working together in a structured partnership, each filling their own natural niche. Business aspects that can sustain the development, validation and maintenance of data access, integration, search and analysis efforts are thus an important challenge in bioinformatics.

The major driving force of the growing bioinformatics market is the need for *new drug development technologies*. Currently, the major pharmaceutical companies are suffering from a lack of revolutionary new ideas, the use of which, in turn, will require new approaches to develop a new generation of drugs. In trying to address the crucial need of bioinformatics methods for understanding disease mechanisms and boosting the drug discovery process, many active players in this market, such as big and middle size pharmaceutical companies, university clinics and governmental institutions, initiated their own plan, thereby producing the same solutions again and again. As a result, the pharmaceutical industry, the only constituency that can "take a drug all the way to the market" invests lesser funds in pursuing in-depth biological studies. Community initiatives in structural public-private partnerships, such as in the IMI programme, may be a solution for sharing costs and granting proper access to data, e.g. core legacy data (Open PHACTS [12]), pharma data (European Translational Information and Knowledge Management Services - ETRIKS [51]), clinical data (European Medical Information Framework - EMIF [52]) and even compounds (European Lead Factory - ELF [53]). New mixed business models for software and data, e.g. based on the *Freemium *[54] model, could guarantee the sustainability of the projects mentioned above.

At the same time, the use of computer technologies and bioinformatics opens new opportunities for drug discovery, research and development, which have not been widely applied until now. Small biotech firms and public institutes could form a rapidly growing force in the early stages of drug development, while "big pharma" industries could likely be more and more specialising in the later phases of lead development and final marketing. Several commercial companies have already been created to fill these needs. These companies offer bioinformatics tools and databases that provide generic solutions for some of the drug development process' tasks. Although such commercial tools are used quite extensively, still the overall annual revenue value of all these companies totals only to about 100 million dollars. This is almost 100 times less than the overall bioinformatics market needs (8.6 billion dollars in 2014, see Table 2), which means that currently most of the money in this field is spent on creating many similar in-house solutions: a really inefficient way of spending resources.

**Table 2:** Global bioinformatics market by submarket.

Segment	2007	2008	2009	2014
Tools	659.10	850.30	1,099.20	4,071.90
Content/database	948.40	1,133.70	1,358.50	3.439.20
Services	222.20	276.50	345.10	1,093.00
**Total**	**1,829.70**	**2,260.50**	**2,802.80**	**8,604.10**

2007-2014 values in million dollars (from: Business Insights, Ltd. report).

In such a situation, the development of **universal bioinformatics platforms **capable of providing unified solutions for drug discovery is urgently needed. Such platforms can only succeed in a public-private partnership setting, or at least with the proper mix of Freemium and highly secure options to serve the needs of all players in all stages of fundamental and translational research. One example of an approach to tackle this challenge is the *BioUML *platform [55] that was developed as an open modular system, consisting of a series of software and databases, which covers most fields of bioinformatics, including modelling, statistics, systems biology and chemoinformatics. It contains many modules, developed by various parties, both on a commercial basis and for a public use. Since researchers are likely to prefer solutions that are adapted to their purposes, including those modules that they get from third parties integrated with their own solutions, BioUML also supports the integration of tools and creation of customized solutions for a particular user. Furthermore, the open source nature of the BioUML platform allows the creation of new modules by the community of third-part developers, thus increasing the number of modules and features in the platform.

BioUML is only one possible solution: there is a chance that other similar efforts develop, thus limiting the efficiency of a unique shared platform. ELIXIR may play a crucial role, in close coordination with other projects and Institutes like, for instance, SageBionetworks, National Center for Biotechnology Information (NCBI) and European Bioinformatics Institute (EBI), in shaping the ecosystem around these major needs, by also ensuring the right balance between huge top down projects and a plethora of academic platforms missing the needed mix of scientific and professional quality.

On the industrial side, companies with a viable bioinformatics expertise in-house may incorporate a set of tools and databases in their own information infrastructure. Others may want to use a safe cloud model or may even outsource their research in this field. A core of legacy data that can be combined with proprietary company data is clearly the future, and the entire range of possibilities, from completely open to completely closed, may therefore be required.

As stated above, bioinformatics is one of the fastest growing segments in the life sciences sector. Bioinformatics and data publishing platforms, such as those described in this position paper and especially the open source based platforms, have a considerable opportunity in this market. We expect that such platforms may awake the interest of several classes of consumers, such as:

•* programmers *developing software for bioinformatics and systems biology, who will get an opportunity for quick and easy creation of various new software modules,

•* bioinformaticians *in large pharmaceutical companies and in academic institutions, who serve the experimental laboratories in support of bioinformatics infrastructures,

•* biologists *and *medical chemists*, who are the end users.

Table 3 lists the main reasons why each of these users will be interested in the platforms. The introduction of such universal platforms will open yet another business opportunity for publishers, biologists and bioinformaticians, namely providing data analysis services through the platforms. Services take a big part of the market and the share of services in the bioinformatics market is growing, mainly due to the fact that major pharmaceutical companies choose to outsource many of the research and development activities. Currently, most of the active service providers are creating their own mix of tools and approaches, leading to many different offerings of solutions that often contradict, or simply do not fit together, in case several steps of the data analysis are outsourced to different providers. Universal, open platforms can solve most of these problems by providing, on one side, a universal interface for all data inputs and outputs and by constituting, on the other side, a free market place for the service providers.

**Table 3:** User classes of open source bioinformatics platforms and main reasons why they will be interested in the platforms.

Users	Reasons to be interested in open source bioinformatics platforms
***Software programmers***	
	Convenient tools and utilities for creating new modules
	Ready to use libraries of classes for working with main bioinformatics and system biology objects (e.g. sequences, genes, networks, etc.)
	Ready integration with all main databases that are needed for working with new modules
	Ability to upload personal modules to the platform and set the policy of their licensing (free, or commercial through an application store)
***Bioinformaticians***	
	Convenient unified environment that combines a variety of programs and algorithms in different ways, which may become necessary for the analysis of different data from laboratory tests
	Unified interface for all modules of the platform that eases the training process of the end users
	Convenient system that can use several programming languages and statistical packages for the creation of scripts, which bioinformaticians can prepare for their further usage in processing of large amounts of routine data
	Convenient system for construction of work procedures for automatic execution of a given sequence of programs; after their creation, the obtained procedures are passed to end users for automated processing of new data
***Biologists and medical chemists***	
	Availability of a large number of ready-to-use modules on different branches of bioinformatics, system biology and computer aided drug modelling
	User-friendly interfaces
	Ability of creation of personalized structured data repository "in the cloud", with data of different origins (e.g. transcriptomics, proteomics, etc.)
	Ability to provide reproducible research
	Ready-to-use operating procedures for automatic execution of given sequences of programs that can answer dedicated biological questions

## Conclusions

Great advances in bio-molecular data production are solving the previous paucity of biological-molecular data; computational standards and techniques are now needed to prevent and reduce the inaccuracy of these data, as well as to support their interpretation. The aim is to provide a quantity of reliable and precise enough data to be used in data driven computational inference and biological knowledge discovery. This transition is rapidly shifting the current main issues in the life sciences towards managing the enormous amount of diverse data effectively and making sense of it. We have mentioned the challenges and how they are tackled in major international initiatives. Other scientific communities, such as physicists, also generate huge amounts of data and have already solved some of the related issues. Yet, challenges are different. To begin with, physics deals with fewer objects than the life sciences; thus, complexity (i.e. number of possible relationships between objects) is greater in the life sciences than in physics, where the same objects always occur in the large physics datasets. Consequently, in the new life science panorama, finding, selecting, extracting, meaningfully integrating and comprehensively processing the most reliable and appropriate information raise numerous issues. Computer science can support their solving in several ways, firstly through formalization and modelling of entities and relationships. Formal modelling provides many critical advantages, including the non-ambiguous definition of entities and concepts, which directly supports integration, search and comprehensive analysis of multiple, heterogeneous and complex bio-data. Secondly, bioinformatics solutions can encompass the standardization of common data and information capture and the interoperability of infrastructures; they can also support data semantics to ease direct integration and comparison with new data.

Based on large and commonly supported research infrastructures, universal computational platforms capable of providing unified solutions for multiple life science needs are emerging. They can both provide universal interfaces for data inputs and outputs. The introduction of such universal platforms represents an additional business opportunity in the fast growing bioinformatics sector of the life science market. This business model, together with open source and open access policies, has the potential to sustain the development and maintenance of good computational systems and effective data integration and validation efforts.

Besides technological and methodological aspects, social aspects are currently playing a very relevant role in bioinformatics and in the life sciences in general. Among them a crucial aspect is the difficulty of attracting contributions to sharing and annotating data, due to inappropriate interfaces and the limited use of adequate provenance and reward models. The actual accessibility and reusability of the data is the main underlying issue that can be addressed with the inclusion of metadata, including rich provenance information as in nanopublications, a recently proposed scheme for publishing a potentially massive number of associations in a "discoverable" and interoperable format.

In conclusion, we strongly recommend that bioinformaticians and experimental scientists first carefully consider joining one of the existing community efforts mentioned in this paper, before deciding to embark on any of these challenges in splendid isolation.

## List of abbreviations used

API: Application Programming Interface; BAM: Binary Alignment/Map; BED: Browser Extensible Data; bigWig: Binary Wiggle; BMC: BioMed Central; BMS: Biological and Medical Sciences; ChEMBL: Chemical European Molecular Biology Laboratory database; ChIP-Seq: Chromatin Immunoprecipitation Sequencing; DAS: Distributed Annotation System; DNA: Deoxy-ribo-Nucleic Acid; DOI: Digital Object Identifier; EBI: European Bioinformatics Institute; EDAM: EMBRACE Data and Methods; ELF: European Lead Factory; EMBRACE: European Model for Bioinformatics Research And Community Education; EMIF: European Medical Information Framework; ESFRI: European Strategic Forum on Research Infrastructures; ETRIKS: European Translational Information and Knowledge management Services; EU: European Union; FASTQ: FASTA Quality; FP6: Framework Programme 6; FP7: Framework Programme 7; IEO-IIT: Istituto Europeo di Oncologia - Istituto Italiano di Tecnologia; IMI: Innovative Medicine Initiative; KBBE: Knowledge-Based Bio-Economy; NCBI: National Center for Biotechnology Information; NGS: Next-Generation Sequencing; Open PHACTS: Open Pharmacological Concept Triple Store; PLoS: Public Library of Science; Polimi: Politecnico di Milano; PubMed: Public/Publisher MEDLINE; RDF: Resource Description Framework; ROC: Receiver Operating Characteristic; SAM: Sequence Alignment/Map; SNP: Single Nucleotide Polymorphism; SPO: subject-predicate-object triple; UML: Unified Modeling Language; UniProt: Universal Protein Resource; URI: Uniform Resource Identifier; UUID: Universally Unique Identifier.

## Competing interests

The authors declare that they have no competing interests. AK works for geneXplain company.

## Authors' contributions

MM and PR conceived the idea behind this work, lead the discussion among authors, and together with FL and BM, coordinated the writing of the sections of the manuscript, other than contributing to write the manuscript.

BM discussed the high relevance of provenance and reward models, together with open exchange and access methodologies, to support motivation and foster personal contributions of experts towards active contribution to data and information integration.

EBR provided the overall view of major recent past and ongoing bio-data and tool integration projects, discussing the most notable of such efforts in human, animal and plant domains.

SC presented and discussed the technological challenges for data integration, exchange and search, providing a view on how to tackle them.

AK gave the business view by presenting and discussing current business models for bioinformatics, which can also allow maintenance of good developed computational systems and universal platforms for the community.

FR illustrated methodological aspects related to data and process formal modelling and how they can contribute to the life sciences.

FL introduced the importance of social aspects involved in effective data integration and validation efforts.

All authors revised the manuscript and read and approved its final version.

## Declarations

Publication of this article has been funded by the IRCCS AOU San Martino IST.

EBR's contribution has been partially supported by COST-BMBS Action BM1006 "Next Generation Sequencing Data Analysis Network", SeqAhead, The ALLBIO project "Broadening the Bioinformatics Infrastructure to unicellular, animal, and plant science", a EU FP7 [Theme: KBBE.2011.3.6-02], Grant agreement no: 289452.

AK's contribution has been partially supported by EU FP7 grants SysCol, MIMomics and BMBF grant GeronotShield.

This article has been published as part of *BMC Bioinformatics *Volume 15 Supplement 1, 2014: Integrated Bio-Search: Selected Works from the 12th International Workshop on Network Tools and Applications in Biology (NETTAB 2012). The full contents of the supplement are available online at http://www.biomedcentral.com/bmcbioinformatics/supplements/15/S1.
